# DSC2 suppresses the growth of gastric cancer through the inhibition of nuclear translocation of γ-catenin and PTEN/PI3K/AKT signaling pathway

**DOI:** 10.18632/aging.204858

**Published:** 2023-07-08

**Authors:** Chao Sun, Kun Wen, Bin Zhang, Yan Dong, Chen Chen, Shi-Yong Neo, Bing Leng, Tian-Tian Gao, Jing Wu

**Affiliations:** 1 Department of Pharmacy, The Second Hospital of Shandong University, Jinan 250033, China; 2 Department of Pharmacy, Shandong Provincial Hospital Affiliated to Shandong First Medical University, Jinan 250021, China; 3 Department of Pharmacy, The First Affiliated Hospital of Shandong First Medical University and Shandong Provincial Qianfoshan Hospital, Jinan 250014, China; 4 Department of Critical Care Medicine, The Second Hospital of Shandong University, Jinan 250033, China; 5 Singapore Immunology Network, Singapore 138648, Singapore

**Keywords:** gastric cancer, desmocollin2, γ-catenin, PTEN/PI3K/AKT

## Abstract

Background: Globally, gastric cancer (GC) is still a major leading cause of cancer-associated deaths. Downregulated desmocollin2 (DSC2) is considered to be closely related to tumor progression. However, the underlying mechanisms of DSC2 in GC progression require further exploration.

Method: We initially constructed different GC cells based on DSC2 contents, established the mouse tumor xenografts, and subsequently performed clonal formation, MTT, Caspase-3 activity, and sperm DNA fragmentation assays to detect the functions of DSC2 in GC growth. Subsequently, we performed western blot, Co-IP, and immunofluorescence assays to investigate the underlying mechanisms through pretreatment with PI3K inhibitor, LY294002, and its activator, recombinant human insulin-like growth factor I (IGF1).

Result: DSC2 could significantly inhibit the viability of GC cells at both *in vitro* and *in vivo* levels. The underlying mechanism may be that DSC2 binds the γ-catenin to decrease its nuclear level, thereby downregulating the anti-apoptotic factor BCL-2 expression and upregulating the pro-apoptotic factor P53 expression, which adjusts the PTEN/PI3K/AKT signaling pathway to promote the cancer cell apoptosis.

Conclusions: Our finding suggests that DSC2 might be a potential therapeutic target for the treatment of cancers, most especially GC.

## INTRODUCTION

Gastric cancer (GC) is a huge threat to the global health and has become the third leading cause of cancer-related death worldwide [1]. The progression of GC involves multiple steps and is considerably complicated involving numerous factors. Despite developmental surgery, radiotherapy, and chemotherapy, the median survival time of GC patients remains short [2]. Therefore, there is an urgent need to further explore the mechanisms of GC progression, find new target biomarkers, and identify more optimal therapeutic schemes for GC patients [3].

Generally, about 90% of malignant tumor cells originate from the epithelium [4]. Among them, GC is mainly derived from the mucosal epithelial cells of the stomach wall. Desmocollins (DSCs) and Desmogleins (DSGs) are transmembrane proteins belonging to the desmosome cadherin family. Both of them play important roles in maintaining epithelial internal environment stability [5]. Therefore, the abnormal expression of DSCs and DSGs might affect cell differentiation, apoptosis, and proliferation [6]. In normal stomach tissue, only the DSC2 was found to be expressed among DSCs subtypes (including DSC1, DSC2, DSC3) [7]. Khan K et al. reported that colorectal adenocarcinoma patients with decreased expression of DSC2 gene had shorter survival time [4]. Knösel T et al. demonstrated that deficiency of DSC2 promoted the proliferation of colorectal cancer cells [7, 8]. Furthermore, aberrant expression of DSC2 had been reported in a pancreatic ductal adenocarcinoma, oral squamous carcinoma and esophageal squamous cell carcinoma [9–11]. In addition, the level of DSC2 was reported to be negatively correlated with lymph node metastasis and pTNM stages in esophageal squamous cell carcinoma [11]. DSC2 also was reported to inhibit the migration and invasion ability of gastric cancer, lung cancer, squamous cell carcinoma of head and neck [12–14], and so on. Cancer Genome Atlas data demonstrate the critical prognostic role of DSC2 in GC. However, the underlying mechanism by which DSC2 drives the progression of GC is still unclear.

In this work, we constructed GC cells with both the downregulation and upregulation of DSC2, established mouse tumor xenograft models, and evaluated the effect of DSC2 on GC cell viability *in vitro* and *in vivo*. The associated mechanisms were detected through pretreatment with PI3K inhibitor, LY294002, and its activator, IGF1, and subsequently performing western blot, co-immunoprecipitation (Co-IP), and immunofluorescence assays. We aimed to establish a novel target to inhibit the growth of GC.

## RESULTS

### DSC2 suppressed the growth of GC both *in vitro* and *in vivo*

**Figure 1:**
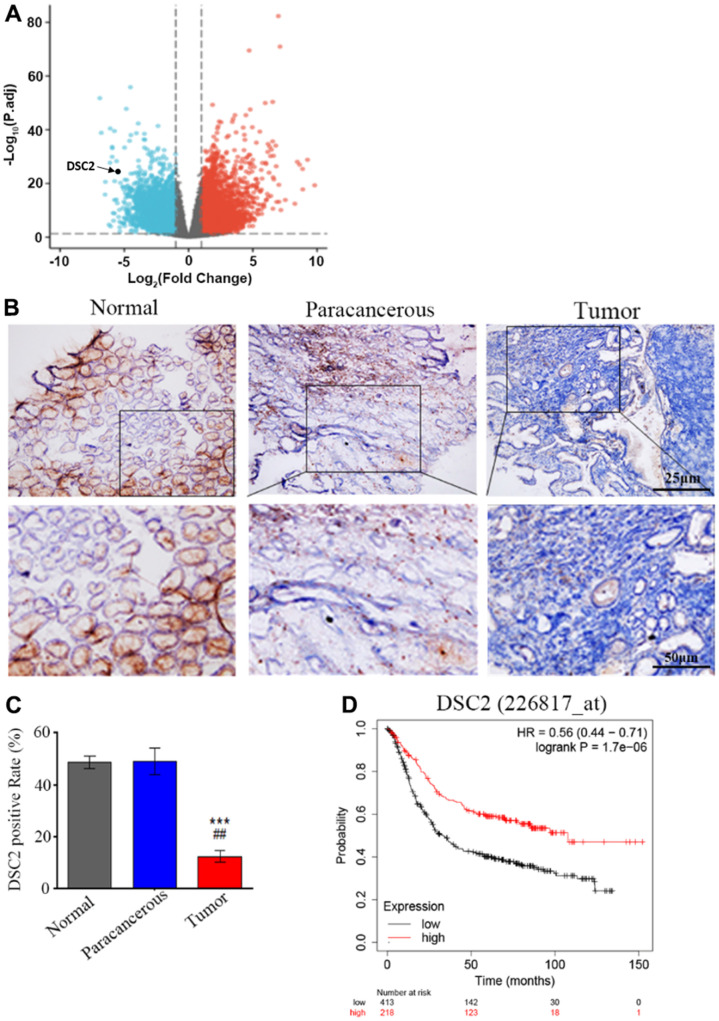
**DSC2 is downregulated in GC tumors and its expression is prognostic for worse overall survival.** (**A**) Volcano plot displayed the pattern of upregulated and downregulated genes in normal and tumor specimens of GC patients from TCGA data, the red dots in the right plot represented upregulated genes, blue dots denoted downregulated genes with statistical significance, and gray dots exhibited no differentially expressed genes. (**B**, **C**) The expressions of DSC2 in adjacent normal tissues (N), paracancerous tissues (P), and GC tissues (T) of clinical specimens were tested through IHC, blue is the nucleus and brownish yellow is the DSC2 expression, the scale bar = 25 μm, ***p<0.001 vs. N, ##p<0.01 vs. P. (**D**) The Kaplan-Meier curves (overall survival) of GC patients with different expression of DSC2, the data was obtained from the website: https://kmplot.com/analysis/index.php?p=service.

**Figure 2:**
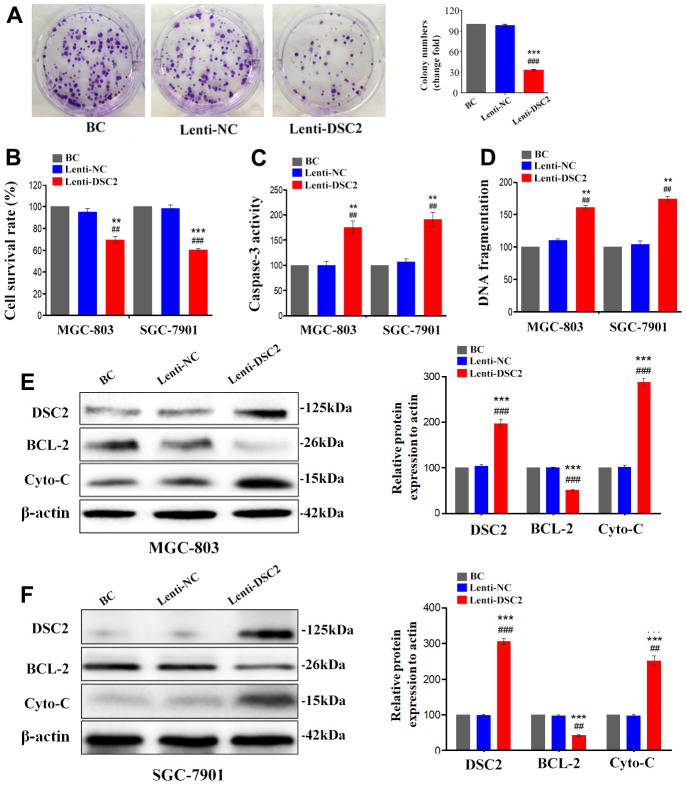
**DSC2 suppressed viability of GC cells *in vitro*.** After stably expressing DSC2 gene, the viability of cells was determined by colony formation assay (**A**, in MGC-803 cells), MTT assay, Caspase-3 activity assay and sperm DNA fragmentation assay (**B**–**D**, in MGC-803 and SGC-7901 cells). (**E**, **F**) The expression of BCL-2 and Cyto-C were determined by Western blot assay. Data are presented as mean ± SEM from three separate experiments. **p<0.01 and ***p<0.001 vs. BC. ##p<0.01 and ###p<0.001 vs. Lenti-NC group. BC: blank control group, Lenti-NC: lentivirus negative control group.

**Figure 3:**
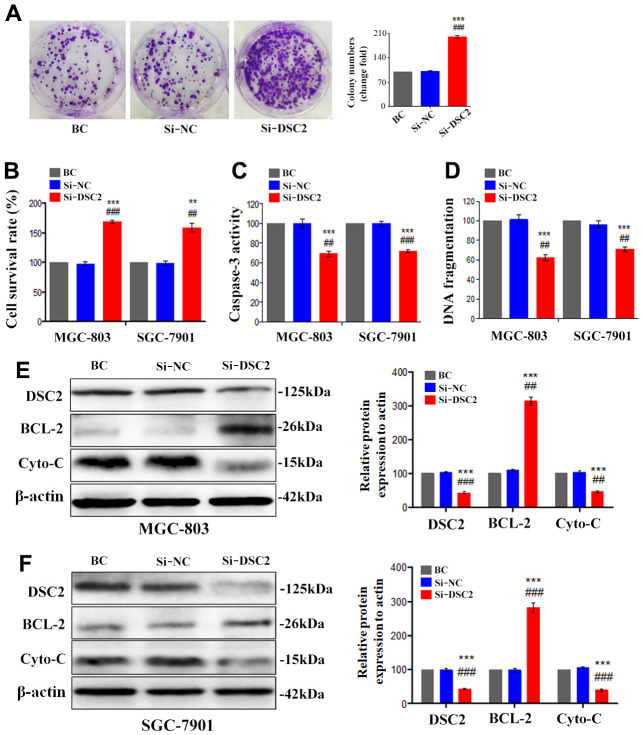
**Silencing the DSC2 gene promoted viability of GC cells *in vitro*.** After being transfected with siDSC2(1+2), the viability of cells was determined by colony formation assay (**A**, in MGC-803 cells), MTT assay, Caspase-3 activity assay and sperm DNA fragmentation assay (**B**–**D**, in MGC-803 and SGC-7901 cells). (**E**, **F**) The expression of BCL-2 and Cyto-C were determined by Western blot assay. Data are presented as mean ± SEM from three separate experiments. **p<0.01 and ***p<0.001 vs. BC. ##p<0.01 and ###p<0.001 vs. Si-NC group. Si-NC: Small interfering negative control group.

We first explored the expression profile of DSC2 in GC patient specimens based on immunohistochemistry. As compared to adjacent normal and paracancerous tissues, DSC2 expression was downregulated in tumors (Figure 1C, 1D). More importantly, volcano plot analysis showed that DSC2 was downregulated in GC tissues than in normal stomach specimens (Figure 1A), and Kaplan-Meier analysis showed that GC patients with high-expression of DSC2 had longer survival time (Figure 1E). Then, we explored the role of DSC2 in the viability of GC cells. Clonal formation, MTT, Caspase-3 activity, and sperm DNA fragmentation assays were used to analyze the role of DSC2 in the viability of GC cells. Overexpression of DSC2 significantly reduced the colony numbers, survival rate of GC cells and correspondingly increased caspase-3 activity and DNA fragmentation level (Figure 2A–2D). Silencing the DSC2 gene had the opposite effect (Figure 3A–3D). Furthermore, suppression of DSC2 resulted in the upregulation in the expression levels of BCL-2 in GC cells, and downregulation in the expression levels of Cyto-C (Figure 3E, 3F). Overexpression of DSC2 had the opposite effects on the expression levels of BCL-2 and Cyto-C in GC cells (Figure 2E, 2F). These data practically demonstrated that DSC2 inhibited the progression of GC, the expression of DSC2 resulted in the inhibition of viability of GC cells, while the downregulation of DSC2 increased the viability of GC cells.

### DSC2 suppressed GC growth *in vivo*

**Figure 4:**
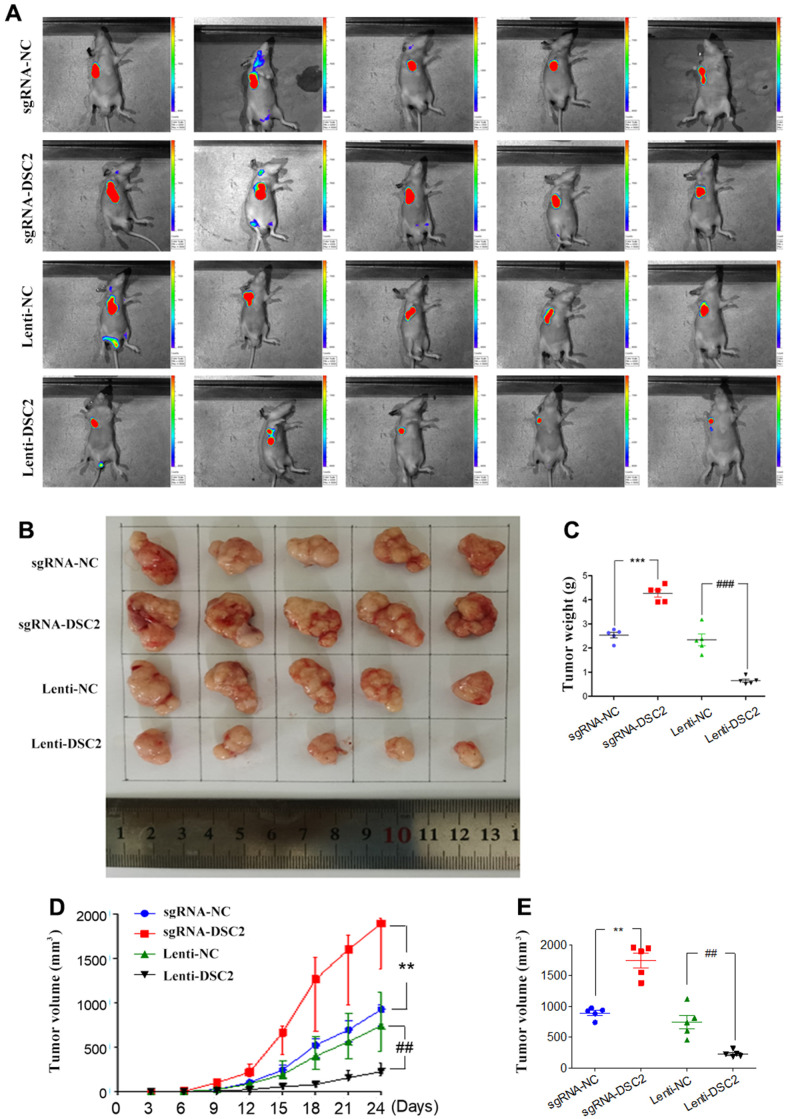
**DSC2 suppressed GC growth *in vivo*.** (**A**) Representative IVIS imaging for scapular bearing tumor. At the day 24, mice were sacrificed and tumors were surgically removed, photoed (**B**), weighed (**C**). Tumor volume of mice were measured every 3 days (**D**), and after removing from the sacrificed mice (**E**). Data are presented as mean ± SEM (n = 5). **p<0.01 and ***p<0.001 vs. sgRNA-NC. ##p<0.01 and ###p<0.001 vs. Lenti-NC.

The effects of DSC2 on the *in vivo* growth of GC were investigated by establishing mice xenograft models of MGC-803 cells. We measured the volumes of xenografts every three days (Figure 4D), while we observed the growth of xenograft tumors with an IVIS Kinetic *in vivo* imaging system at day 24 (Figure 4A). Subsequently, the xenografts were removed from the mice, the tumor were photographed (Figure 4B), the volumes were measured (Figure 4E) and the xenografts were weighed (Figure 4C). The xenograft tumor volumes and weights of the sgRNA-DSC2 group were markedly larger than those in the sgRNA-NC group. On the contrary, the overexpression of DSC2 dampens tumor growth *in vivo* whereby xenograft tumor sizes in the Lenti-DSC2 group were significantly smaller than those in the lenti-NC group. The tumor weights in the sgRNA-NC group, sgRNA-DSC2 group, lenti-NC group, and Lenti-DSC2 group were (2.43 ± 0.33) g, (4.29 ± 0.38) g, (2.45 ± 0.73) g, and (0.70 ± 0.19) g, respectively. These data suggest that the upregulation in DSC2 expression levels significantly inhibited the primary tumor growth of MGC-803 cells in nude mice, indicating that DSC2 suppressed tumor growth *in vivo*.

### DSC2 suppressed the viability of GC cells by inhibiting the nuclear translocation of γ-catenin through the formation of a DSC2/γ-catenin complex

**Figure 5:**
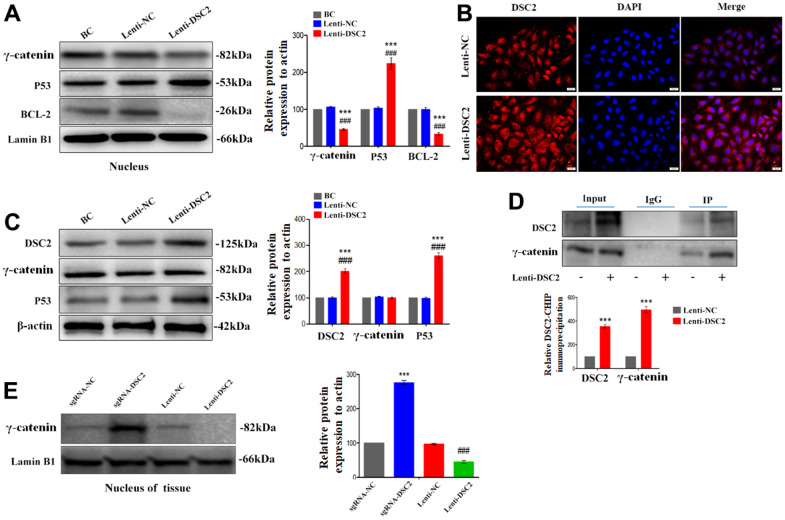
**DSC2 inhibited the γ-catenin nuclear translocation of MGC-803 cells by forming DSC2/γ-catenin complex.** After stably expressing DSC2 gene of MGC-803 cells, (**A**) the expression levels of γ-catenin, BCL-2 and P53 in nucleus was determined by Western blot assay. (**B**) The level of γ-catenin accumulated in the nucleus was detected by immunofluorescence assay. The scale bar = 20 μm. (**C**) The expressions of γ-catenin and P53 were detected by Western blot assay. (**D**) Co-IP assay was performed to analyse the interaction between DSC2/γ-catenin by DSC2. The data are represented as mean ± SEM, n=3. ***p<0.001 vs. BC. ###p<0.001 vs. Lenti-NC. (**E**) The expression of γ-catenin in the nucleus of tumor xenograft tissues were detected by Western blot assay. Data are presented as mean ± SEM, n=5. ***p<0.001 vs. sgRNA-NC. ###p<0.001 vs. Lenti-NC.

**Figure 6:**
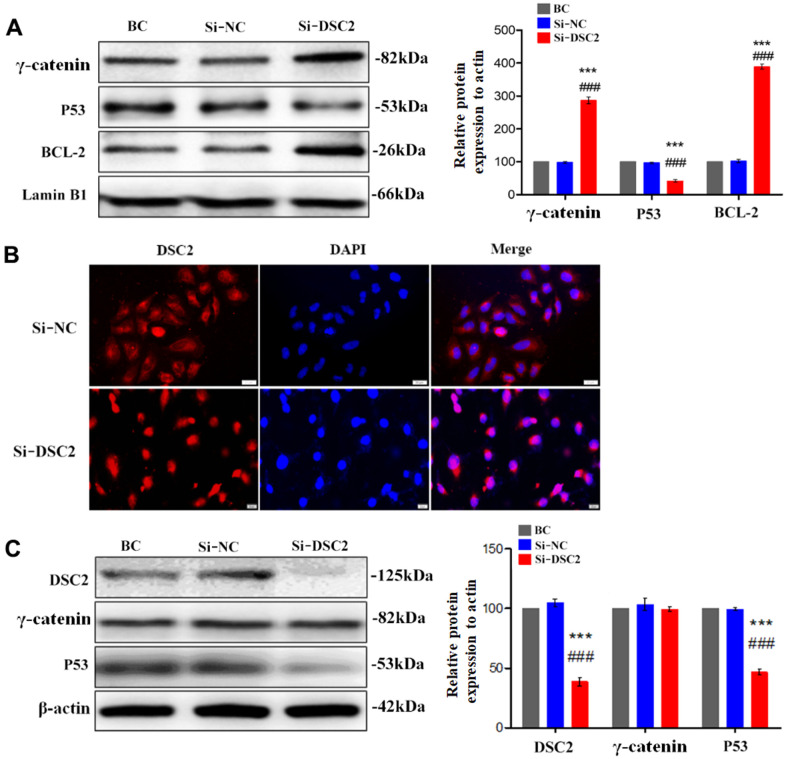
**Downregulation of DSC2 expression promoted the γ-catenin nuclear translocation of MGC-803 cells.** After being transfected with siDSC2 in MGC-803 cells, (**A**) the expression level of γ-catenin, BCL-2 and P53 in nucleus was determined by Western blot assay. (**B**) The distribution of γ-catenin in cells was detected by immunofluorescence assay. The scale bar = 20 μm. (**C**) The expressions of γ-catenin and P53 were detected by Western blot assay. The data are represented as mean ± SEM, n=3. ***p<0.001 vs. BC. ###p<0.001 vs. Si-NC.

As an important component of functional desmosomes, γ-catenin links with DSC2 in cytoplasm. γ-catenin is an important transcription regulatory factor, which mediates the transcription of target genes after translocating to the nucleus, and plays important role in inhibiting the growth of GC [15]. We performed liquid chromatography-tandem mass spectrometry (LC-MS/MS), Co-IP, Western blot, and immunofluorescence staining assays to detect the levels of γ-catenin that combined with DSC2 and the nuclear translocation of γ-catenin. The results showed that γ-catenin was an interactor for DSC2 in GC cells (Supplementary Table 1) [14]. Upon the overexpression of DSC2 in GC cells, the levels of γ-catenin combined with DSC2 were increased (Figure 5D and Supplementary Figure 2A) [14], while both *in vitro* and *in vivo* expression levels of γ-catenin in the nucleus were reduced (Figure 5A, 5B, 5E and Supplementary Figure 2B, 2D). Furthermore, the suppression of DSC2 correspondingly increased the nuclear translocation of γ-catenin (Figure 6A, 6B and Supplementary Figure 2B, 2C).

**Figure 7:**
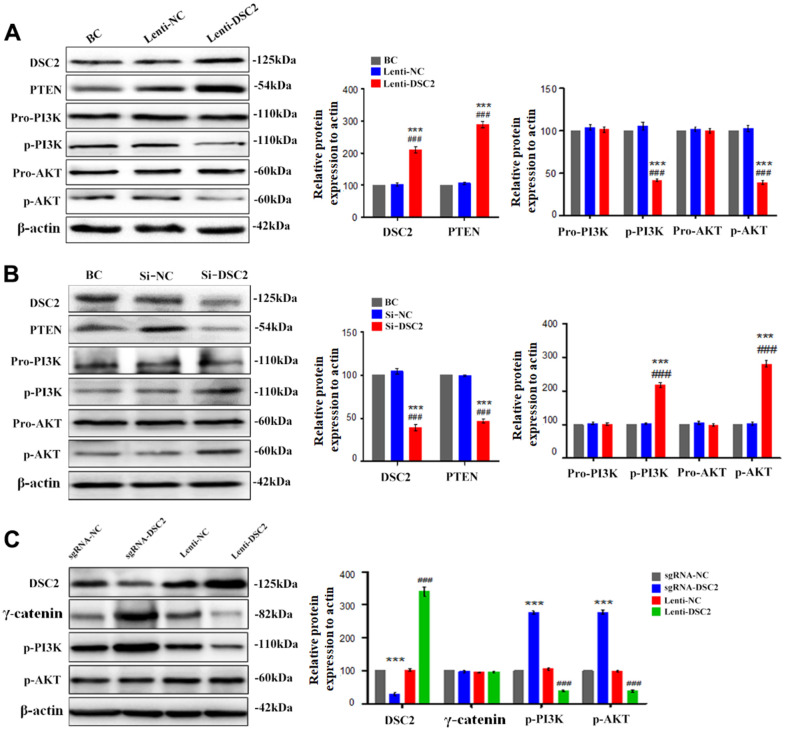
**DSC2 inhibited the PI3K/AKT signaling pathway in MGC-803 cells.** After stably expressing and knocking down DSC2 gene in MGC-803 cells, (**A**, **B**) the expressions of PTEN, pro-PI3K, p-PI3K, pro-AKT, p-AKT were detected by Western blot assay. Data are presented as mean ± SEM from three separate experiments. ***p<0.001 vs. BC. ###p<0.001 vs. Si-NC or Lenti-NC. (**C**) The expressions of DSC2, γ-catenin, p-PI3K and p-AKT in tumor xenograft tissues were detected by Western blot assay. Data are presented as mean ± SEM, n=5. ***p<0.001 vs. sgRNA-NC. ###p<0.001 vs. Lenti-NC.

BCL-2 and p53 are the downstream signals of γ-catenin and are associated with cell viability. Our results showed that overexpression of DSC2 downregulated BCL-2 and upregulated p53 in the nuclei of GC cells (Figure 5A and Supplementary Figure 2D), while the suppression of DSC2 exhibited the opposite effects (Figure 6A and Supplementary Figure 2C). Interestingly, neither the suppression nor overexpression of DSC2 regulated the total levels of γ-catenin in either of the GC cells or mouse tumor xenografts (Figures 5C, 6C, 7C and Supplementary Figure 2E, 2F). These findings suggest that DSC2 induced the formation of a DSC2/γ-catenin complex. The complex inhibited γ-catenin dissolution and nuclear translocation, resulting in the regulation of the transcription of target genes and the suppression of GC cell viability. DSC2 did not affect both the *in vitro* and *in vivo* expression levels of γ-catenin, but merely induced the intracellular redistribution of γ-catenin.

### DSC2 downregulated the PTEN/PI3K/AKT pathway to inhibit the viability of GC cells

The level of p53 in the nucleus was positively correlated with the expression of DSC2 in the GC cells (Figures 5A, 6A). P53 is the transcriptional regulator of PTEN; PTEN is reported as a key regulator of the PI3K/AKT signaling pathway in certain cancers [9]; and the activation of the PI3K/AKT pathway is involved in regulating the viability of GC cells [16]. We detected the effects of DSC2 on the PTEN/PI3K/AKT signaling pathway in GC cells. PTEN and p53 expression levels were both positively correlated with the expression of DSC2 in GC cells (Figures 5C, 6C, 7A, 7B and Supplementary Figure 2E, 2F). The overexpression of DSC2 in MGC-803 and SGC-7901 cells markedly decreased the expression levels of p-PI3K and p-AKT, while the suppression of DSC2 markedly increased the expression levels of these proteins (Figure 7A, 7B and Supplementary Figure 2E, 2F). These findings were also observed in transplanted tumor tissues in nude mice (Figure 7C).

**Figure 8:**
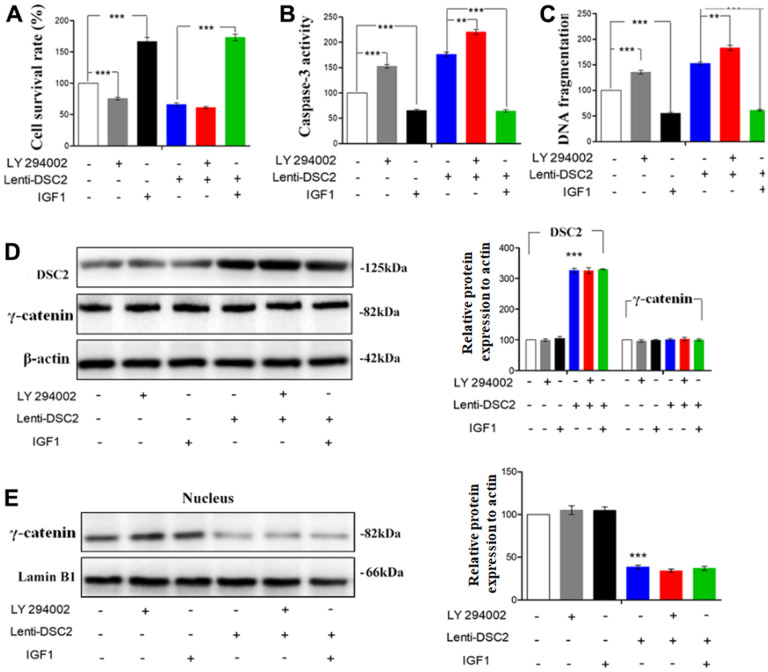
**DSC2 inhibited the viability of MGC-803 cells through suppressing PI3K/AKT/mTOR signaling pathway.** Effect of DSC2 on the viability of GC cells in the presence of LY294002 and IGF1 was determined by Caspase-3 activity assay (**A**), Sperm DNA fragmentation assay (**B**) and MTT assay (**C**). Data are presented as mean ± SEM from three separate experiments. **p<0.01 and ***p<0.001 vs. Lenti-NC or Lenti-DSC2 group. (**D**, **E**) The levels of γ-catenin both in cells and in the nucleus among MGC-803 cells that treated with LY294002 or IGF1, were tested by Western blot assay. The data are represented as mean ± SEM, n=3. ***p<0.001 vs. Lenti-NC.

To further investigate the effects of DSC2 on the PI3K/AKT signaling pathway in GC cells, MGC-803 and SGC-7901 cells were pretreated with PI3K inhibitor, LY294002 (30 μM), and its activator, IGF1 (20 μM), for 24 h or 48 h. Then, MTT, Caspase-3 activity, and sperm DNA fragmentation assays were performed. Quantitative data showed that overexpression of DSC2 increased caspase-3 activity and DNA fragmentation levels in GC cells, with the attendant inhibition of cell viability. LY294002 markedly enhanced caspase-3 activity and DNA fragmentation levels in Lenti-DSC2 GC cells. However, IGF1 dramatically reversed the inhibitory effect of DSC2 on the survival rate of Lenti-DSC2 GC cells, accompanied by reversal of the increased caspase-3 activity and DNA fragmentation level in GC cells as induced by DSC2 (Figure 8A–8C and Supplementary Figure 3A–3C). These results demonstrated that PI3K activation reversed the proliferation-inhibiting effects of DSC2 in GC cells. Moreover, neither the PI3K inhibitor nor its activator affected the expression and nuclear translocation of γ-catenin (Figure 8D, 8E, and Supplementary Figure 3D, 3E). These findings suggest that DSC2 inhibited the viability of GC cells via the regulation of the PI3K/AKT signaling pathway. In DSC2-deficient GC cells, the increased nuclear translocation and transcriptional activity of γ-catenin were not related to the PI3K/AKT signaling pathway.

## DISCUSSION

**Figure 9:**
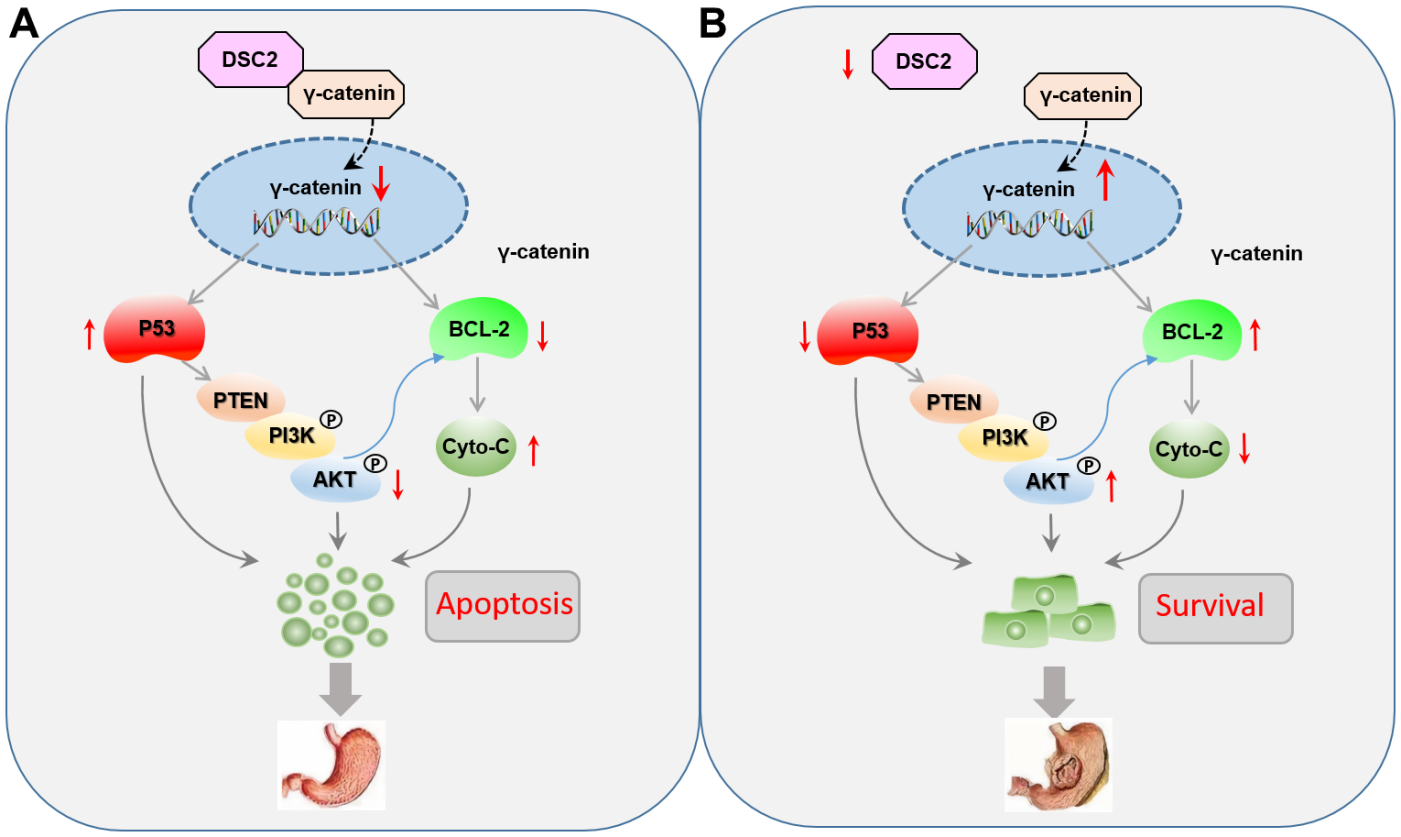
**Role of DSC2 in the survival of gastric cancer cells.** (**A**) DSC2 binds the γ-catenin to decrease its nuclear level, thereby downregulating BCL-2 expression, which adjusts the expression of Cyto-C negatively, and upregulating P53 expression, which adjusts the PTEN/PI3K/AKT signaling pathway to promote the GC cell apoptosis. (**B**) Dissociated γ-catenin translocates to the nuclei of GC cells when DSC2 is destroyed or suppressed, thereby upregulating BCL-2 expression, which suppresses the expression of Cyto-C, and downregulating P53 expression, which adjusts the PTEN/PI3K/AKT signaling pathway to promote the GC cell survival.

GC is a malignant tumor with mucosa epithelial origin driven by complicated molecular processes [17]. Among the several mechanisms known, the desmosome junction is one of the important intercellular junctions to maintain epithelial morphogenesis [7]. As one of the most important components of the desmosome junction, DSCs play a key role in maintaining epithelial cell adhesion, mechanical force, and internal environment stability. Abnormal expression levels of DSCs have been found in urothelial carcinoma, colorectal cancer, and pancreatic ductal adenocarcinoma [18–20]. Thus far, DSC2 is the only subtype among DSC1–3 that has been detected in the stomach tissues. The role of DSC2 in GC growth is still unclear and need to further exploration. However, in the present study, we found that the upregulation of DSC2 suppressed the *in vitro* and *in vivo* viability of MGC-803 and SGC-7901 cells. Recovered DSC2 in GC cells did not only significantly suppress the nuclear translocation of γ-catenin, but also inhibited the PTEN/PI3K/AKT signaling pathway (Figure 9).

γ-Catenin is a transcriptional regulatory factor belongs to the member of the Armadillo (ARM) family. γ-catenin always accumulate in the nucleus and mediates the transcription of target genes, including viability-associated biomarkers [4, 8, 20, 21]. As an important component of functional desmosomes, γ-catenin connects with DSC2 in the cytoplasm to form a complex anchor which binds filaments to the cell membrane. The complexes of DSC2/γ-catenin form a supracellular scaffold and hamper mechanical stress, contributing to the maintenance of intercellular adhesion and epithelial tissue stability [22, 23]. We speculated that the formation of a DSC2/γ-catenin complex may keep free γ-catenin at very low concentrations in the cells, while dissociative γ-catenin from the complex could translocate to the nuclei of GC cells when DSC2 is destroyed or suppressed. In the present study, we found that DSC2 inhibited the nuclear translocation of γ-catenin, while downregulation of DSC2 promoted its nuclear translocation, without affecting the total level of γ-catenin in GC cells. The data repositories of Biological General Repository for Interaction Datasets (BioGRID) and LC-MS/MS assay were used to predict protein-protein interaction for DSC2. Results showed that γ-catenin might be a protein linked with DSC2 (Supplementary Table 1). We deduced that DSC2 induced the formation of a DSC2/γ-catenin complex, inhibited the dissolution of γ-catenin, and suppressed the nuclear translocation of free γ-catenin. Furthermore, γ-catenin expression in the nucleus was positively associated with BCL-2 but negatively associated with important tumor suppressors, such as p53 and PTEN. γ-Catenin is an important transcription regulatory factor that mediates the transcription of target genes such as LEF/TCF in the nucleus, and plays the important role in inhibiting the growth of GC [15, 24]. BCL-2 and P53 are considered to be the target genes of γ-catenin [25]. So we concluded that when the DSC2 was destroyed, free γ-catenin were translocated into the nucleus and then regulated the transcription of viability-associated genes to promote GC growth (Figure 9).

The PTEN/PI3K/AKT signaling pathway is one of the most frequently dysregulated pathways in the development of GC, which was obviously suppressed via the inactivation of this pathway [26, 27]. Kolegraff et al. demonstrated that DSC2 downregulation contributed to the proliferation and metastasis of colorectal cancer cells by activating the AKT/β-catenin signaling pathway [8]. Here, we found that the suppression of DSC2 resulted in the upregulation of the PI3K/AKT signaling pathway and downregulation in p53 and PTEN levels, while the overexpression of DSC2 reversed these regulatory effects. These findings indicated that the levels of PTEN was positively correlated with DSC2 expression, which was consistent with RNA-Seq data. P53 and PTEN are important tumor suppressors and could suppress the PI3K/AKT signaling pathway [28]. In the present study, the PI3K activator significantly reversed the inhibitory effects of DSC2 on the viability of GC cells, while the PI3K inhibitor exhibited no obvious effects. Furthermore, we found that neither the PI3K inhibitor nor its activator had any effects on DSC2 expression, indicating that the PI3K/AKT signaling pathway is a downstream signal of DSC2. In addition, neither the PI3K inhibitor nor its activator had any effects on the expression levels and nuclear translocation of γ-catenin. Therefore, we deduced that the PI3K/AKT signaling pathway is involved in GC progression when DSC2 is deficient, while it does not regulate the transcriptional activity of γ-catenin.

Unfortunately, there were still some limitations in this work. We were unable to find out the specific transcriptional activity inhibitor of γ-catenin. We were not yet able to verify the direct regulatory effect of γ-catenin on the expression of BCL2 and P53. So we just inferred from the experimental results that DSC2 suppressed the nuclear translocation and transcriptional activity of γ-catenin, then inhibited the growth of GC through downregulating the expression of BCL-2 and inhibiting the P53/PTEN/PI3K/AKT signaling pathway, respectively. In the future study, we will continue to search for suitable transcriptional inhibitor of γ-catenin and perform further research.

## CONCLUSIONS

Here, we identified that DSC2 functioned as a pivotal tumor suppressor in GC by inhibiting the nuclear translocation of γ-catenin and inactivating the PTEN/PI3K/AKT signaling pathway. Our finding suggests that DSC2 might be a potential therapeutic target for the treatment of cancers, most especially GC.

## MATERIALS AND METHODS

### Materials

LY294002 was purchased from Beyotime Institute of Biotechnology (Jiangsu, China), while recombinant human insulin-like growth factor I (IGF1) was purchased from R&D Systems (Minnesota, USA).

### Cell culture and collection of tissue samples

Human GC cell lines, MGC-803 and SGC-7901, were kindly provided by the School of Basic Medical Sciences, Shandong University, Jinan, China. SGC-7901 cells were cultured in RPMI-1640 medium, while MGC-803 cells were cultured in DMEM medium. Both media were supplemented with 10% fetal bovine serum (Gibco, CA, USA) and 100 μg/μL streptomycin/penicillin with the cells cultured in a humidified 5% CO2 incubator at 37° C.

### Cell transfection-silenced DSC2 gene

Cells were dispensed into 6-well culture plates and grew to 60%–70% confluence. Transfection with 8 μL DSC2 siRNA-1 and DSC2 siRNA-2 was performed using 8 μL Lipofectamine 2000 Reagent in 500 μL Opti-MEM culture medium (siDSC2 group). The coding strand of DSC2 siRNA-1 was 5ʹ-CUUUACAGCUGCAAAUCUATTUAGAUUUGCAGCUGUAAAGTT-3ʹ, DSC2 siRNA-2 was 5ʹ-CUGGAGAUGACAAAGUGUATTUACACUUUGUCA UCUCCAGTT-3ʹ, and the negative control coding strand (NC group) was 5ʹ-UUCUCCGAACGUGUCACGUTTACGUGACACGUUCGGAGAATT-3ʹ. After incubation for 6 h, the Opti-MEM culture medium was replaced with culture medium with 10% fetal bovine serum (without antibiotics). Then, the cells were incubated in a 5% CO2 incubator at 37° C for 48 h.

### CRISPR/Cas9-induced knockout of the DSC2 gene

Designed primers with NM_024422.6, then constructed sgRNA vectors targeting the human DSC2 gene. gRNA Sequence (target exon 1+2), DSC2-gRNA 1: GGCTAG ATGGTGTCTATACATGG, DSC2-gRNA 2: CACTTAGACATTTCTAGACAAGG. Reaction conditions: 5 min at 94° C, 10 min at 70° C, 10 min at 55° C, and 10 min at 40° C. Subsequently, the vectors of sgRNA-DSC2 or Cas9 were inserted into the CRISPR EGFP plasmid. Then, cells (2 × 10^5^ cells per well) were seeded into 6-well plates. After transfecting with CRISPR DSC2-EGFP plasmid for 48 h, EGFP-positive cells were sorted. After six weeks, the clones were collected and the knockout of DSC2 expression (sgRNA-DSC2 group) was detected by photo and qRT-PCR (Supplementary Figure 1). Negative control cells (sgRNA-NC group) were transfected with the empty vector.

### Stable overexpression of human DSC2 gene

Cells (2×10^5^ cells per well) were seeded into 6-well plates. After transfecting with LV-EFS promoter>hDSC2[NM_024422.6]-CMV>EGFP/T2A/Puro plasmid for 48 h, EGFP-positive cells (Lenti-DSC2 group) were sorted. Negative control cells (lenti-NC group) were transfected with the vector of LV-CMV>EGFP/T2A/Puro.

### Western blot analysis

Cells or tissues were lysed in RIPA buffer and protein concentration was determined by BCA protein assay. Nuclear extraction from total lysis was performed using Subcellular Structure Cytoplasmic and Nuclear Extraction Kit. Then, proteins were separated by 10% sodium dodecyl sulfate-polyacrylamide gel electrophoresis (SDS-PAGE). After electrophoresis, the proteins were electrotransferred to PVDF membrane (Millipore, Billerica, USA) and then blocked with 5% non-fat milk for 4 h. The primary antibodies against DSC2 (#53485, Santa Cruz Biotechnology, USA), p-PI3K (#0854, ABclonal Technology, Wuhan, China), PI3K (#4255, Cell Signaling Technology, Massachusetts, USA), p-AKT (#66444, Proteintech, Wuhan, China), Akt (#60203, Proteintech, Wuhan, China), Cyto-C (#4280, Cell Signaling Technology, Massachusetts, USA), caspase-3 (#66470, Proteintech, Wuhan, China), BCL-2 (#12789, Proteintech, Wuhan, China), p53 (#AF0879, Affinity Biotechnology, USA), PTEN (#60300, Proteintech, Wuhan, China), γ-catenin (#66445, Proteintech, Wuhan, China), Lamin B1 (internal reference for the nucleus, #AF5161, Affinity Biotechnology, USA), and β-actin (internal reference for the total cell, #ZF-0313, ZS Bio, Beijing, China) were used.

### Co-immunoprecipitation (Co-IP)

Cells of each group were lysed in lysis buffer, and the supernatants were removed after centrifugation. Half of the supernatant was taken as the input sample. The remainder was used as the IP sample. Antibodies conjugated with magnetic protein G beads (Santa Cruz Biotechnology, Beijing, China) were added to the IP samples for an overnight incubation at 4° C. Subsequently, the immunocomplexes of DSC2/γ-catenin were recovered and boiled. Thereafter, the expressions of associated proteins were detected by western blot.

### MTT assay

Cells from each group (3-4 × 10^3^ cells per well) were dispensed into 96-well plates and incubated for 24 h. After washing with PBS, cells were incubated in culture medium containing 20 μL of MTT solution (5 mg/mL in phosphate-buffered saline; PBS) for a further 4 h at 37° C. The medium was removed and replaced with 150 μL of DMSO to dissolve the formazan crystal. The absorbance was measured at 570 nm using Thermo Multiskan GO microplate reader (Thermo-1510, CA, USA).

### Sperm DNA fragmentation assay

Cells from each group were labeled with 5-bromodeoxyuridine (BrdU), and then lysed in incubation buffer. The supernatant was collected after centrifugation and treated with anti-DNA antibody coated Quantikine ELISA kits (R&D Systems, MN, USA) overnight at 4° C. In brief, 100 μL of supernatant from each well was added into the Quantikine ELISA kits, and cultured at room temperature for 90 min. The DNA was denatured by heating with medium fire for 5 min to expose BrdU for easy detection. Then, the samples were incubated with 100 μL of anti-BrdU-POD antibody per well at room temperature for 90 min. We added 100 μL tetramethylbenzidine (TMB) per well, and the absorbance was detected at 450 nm with a Thermo Multiskan GO microplate reader (Thermo-1510, CA, USA).

### Caspase-3 activity assay

Cells from each group were lysed in lysis buffer. Then, the protein concentration was determined by Bradford protein assay. Protein (150 μg), 10 μL caspase-3 substrate (5 μM Ac-DEVD-AMC), and 10 μL caspase-3 inhibitor (1 μM Ac-DEVD-CHO) were mixed and seeded into 96-well plates at 37° C for 1 h. The absorbance was determined at 355 nm using the Microplate Luminometer (LB960, Berthold, German).

### Immunofluorescence staining

Cells, from each group, seeded into 24-well plates with a coverslip per well were subsequently fixed in 4% paraformaldehyde prepared with PBS at room temperature for 20 min, and permeabilized with PBS for 30 min, then incubated with DSC2 antibody at 4° C overnight. Subsequently, the cells were washed with PBS and incubated with secondary antibody at room temperature for 1 h. After that, cells were labeled with Hoechst 33342 to stain the nuclei, and fluorescence images were taken with a fluorescence microscope (NIKON Ti-U, Nikon, Japan).

### Mouse tumor xenograft and IVIS imaging

Five-week-old BALB/c nude mice were raised under sterile conditions. All mice were purchased from the Animal Center of China Academy of Medical Science (Beijing, China). All animal experimental procedures conformed to the Institutional Guidelines of Animal Care and Use Committee of Shandong University. The mice were divided randomly into four groups (n = 5 per group): sgRNA-NC group, sgRNA-DSC2 group, lenti-NC group, and Lenti-DSC2 group.

MGC-803 cells (1 × 10^7^) from each group were inoculated to the scapular region of each nude mouse. The mouse weights and tumor sizes were measured every three days. The fluorescence signals of xenografts in all mice were taken with the IVIS Kinetic *in vivo* imaging system at day 24. Then, all mice were euthanized and the xenografts were surgically removed and weighed. Both the long and short axes of removed tumors were determined by vernier caliper measurements. The formula V = L × W2 / 2 was used to determine the tumor volume (V); W refers to the short axis and L refers to the long axis.

### Liquid chromatography-tandem mass spectrometry (LC-MS/MS) assay

Extracted the protein of MGC-803 cells in lenti-NC or Lenti-DSC2 group. Antibodies conjugated with magnetic protein G beads (Santa Cruz Biotechnology, Beijing, China) were added to the samples for an overnight incubation at 4° C. Subsequently, the samples were recovered and boiled. Then, immunoprecipitation elution was loaded onto an SDS-PAGE gel for the next phase of protein separation. Gel pieces were cut from SDS-PAGE and dimensional gel was excised, trypsin-digested, and desalted. Each fraction was injected for nano LC-MS/MS analysis. The instrument was run with the peptide recognition mode enabled. Spectra were searched using the MASCOT engine (Matrix Science, London, UK; version 2.2) against a nonredundant International Protein Index arabidopsis sequence database v3.85 from the European Bioinformatics Institute (http://www.ebi.ac.uk/).

### Statistical analysis

Unless stated otherwise, all quantitative data are expressed as mean ± SEM. Statistical comparisons were performed by one-way analysis of variance. A P-value < 0.05 was considered statistically significant. Statistical analysis was performed using the SPSS/Win 16.0 software. Kaplan-meier survival analysis was perform on “KM-plotter” (accessed on: https://kmplot.com/analysis/index.php?p=service).

### Consent for publication

All the authors agree the names of orders and agree to transfer the copy right to Aging-US.

### Availability of data and material

The data used and/or analyzed during this study are available from the corresponding author upon request.

## Supplementary Material

Supplementary Figures

Supplementary Table 1

## References

[1] Sung H, Ferlay J, Siegel RL, Laversanne M, Soerjomataram I, Jemal A, Bray F. Global Cancer Statistics 2020: GLOBOCAN Estimates of Incidence and Mortality Worldwide for 36 Cancers in 185 Countries. CA Cancer J Clin. 2021; 71:209–49. 10.3322/caac.21660 33538338

[2] Wei L, Sun J, Zhang N, Zheng Y, Wang X, Lv L, Liu J, Xu Y, Shen Y, Yang M. Noncoding RNAs in gastric cancer: implications for drug resistance. Mol Cancer. 2020; 19:62. 10.1186/s12943-020-01185-7 32192494 PMC7081551

[3] Joshi SS, Badgwell BD. Current treatment and recent progress in gastric cancer. CA Cancer J Clin. 2021; 71:264–79. 10.3322/caac.21657 33592120 PMC9927927

[4] Khan K, Hardy R, Haq A, Ogunbiyi O, Morton D, Chidgey M. Desmocollin switching in colorectal cancer. Br J Cancer. 2006; 95:1367–70. 10.1038/sj.bjc.6603453 17088906 PMC2360607

[5] Lowndes M, Rakshit S, Shafraz O, Borghi N, Harmon RM, Green KJ, Sivasankar S, Nelson WJ. Different roles of cadherins in the assembly and structural integrity of the desmosome complex. J Cell Sci. 2014; 127:2339–50. 10.1242/jcs.146316 24610950 PMC4021477

[6] Huber O, Petersen I. 150th Anniversary Series: Desmosomes and the Hallmarks of Cancer. Cell Commun Adhes. 2015; 22:15–28. 10.3109/15419061.2015.1039642 26133535

[7] Sun C, Wang L, Yang XX, Jiang YH, Guo XL. The aberrant expression or disruption of desmocollin2 in human diseases. Int J Biol Macromol. 2019; 131:378–86. 10.1016/j.ijbiomac.2019.03.041 30851326

[8] Kolegraff K, Nava P, Helms MN, Parkos CA, Nusrat A. Loss of desmocollin-2 confers a tumorigenic phenotype to colonic epithelial cells through activation of Akt/β-catenin signaling. Mol Biol Cell. 2011; 22:1121–34. 10.1091/mbc.E10-10-0845 21325624 PMC3078068

[9] Xin Z, Yamaguchi A, Sakamoto K. Aberrant expression and altered cellular localization of desmosomal and hemidesmosomal proteins are associated with aggressive clinicopathological features of oral squamous cell carcinoma. Virchows Arch. 2014; 465:35–47. 10.1007/s00428-014-1594-6 24849508

[10] Hamidov Z, Altendorf-Hofmann A, Chen Y, Settmacher U, Petersen I, Knösel T. Reduced expression of desmocollin 2 is an independent prognostic biomarker for shorter patients survival in pancreatic ductal adenocarcinoma. J Clin Pathol. 2011; 64:990–4. 10.1136/jclinpath-2011-200099 21725043

[11] Fang WK, Gu W, Li EM, Wu ZY, Shen ZY, Shen JH, Wu JY, Pan F, Lv Z, Xu XE, Huang Q, Xu LY. Reduced membranous and ectopic cytoplasmic expression of DSC2 in esophageal squamous cell carcinoma: an independent prognostic factor. Hum Pathol. 2010; 41:1456–65. 10.1016/j.humpath.2010.04.003 20621329

[12] Brown L, Wan H. Desmoglein 3: a help or a hindrance in cancer progression? Cancers (Basel). 2015; 7:266–86. 10.3390/cancers7010266 25629808 PMC4381258

[13] Overmiller AM, McGuinn KP, Roberts BJ, Cooper F, Brennan-Crispi DM, Deguchi T, Peltonen S, Wahl JK 3rd, Mahoney MG. c-Src/Cav1-dependent activation of the EGFR by Dsg2. Oncotarget. 2016; 7:37536–55. 10.18632/oncotarget.7675 26918609 PMC5122330

[14] Sun C, Wang L, Du DD, Ji JB, Yang XX, Yu BF, Shang PF, Guo XL. DSC2 Suppresses the Metastasis of Gastric Cancer through Inhibiting the BRD4/Snail Signaling Pathway and the Transcriptional Activity of *β*-Catenin. Oxid Med Cell Longev. 2022; 2022:4813571. 10.1155/2022/4813571 36120591 PMC9473342

[15] Wickline ED, Du Y, Stolz DB, Kahn M, Monga SP. γ-Catenin at adherens junctions: mechanism and biologic implications in hepatocellular cancer after β-catenin knockdown. Neoplasia. 2013; 15:421–34. 10.1593/neo.122098 23555187 PMC3612914

[16] Nagel JM, Lahm H, Ofner A, Göke B, Kolligs FT. γ-Catenin acts as a tumor suppressor through context-dependent mechanisms in colorectal cancer. Int J Colorectal Dis. 2017; 32:1243–51. 10.1007/s00384-017-2846-0 28681073

[17] Deng C, Zhang L, Ma X, Cai S, Jia Y, Zhao L. RFTN1 facilitates gastric cancer progression by modulating AKT/p38 signaling pathways. Pathol Res Pract. 2022; 234:153902. 10.1016/j.prp.2022.153902 35490655

[18] Hayashi T, Sentani K, Oue N, Anami K, Sakamoto N, Ohara S, Teishima J, Noguchi T, Nakayama H, Taniyama K, Matsubara A, Yasui W. Desmocollin 2 is a new immunohistochemical marker indicative of squamous differentiation in urothelial carcinoma. Histopathology. 2011; 59:710–21. 10.1111/j.1365-2559.2011.03988.x 22014052

[19] Knösel T, Chen Y, Hotovy S, Settmacher U, Altendorf-Hofmann A, Petersen I. Loss of desmocollin 1-3 and homeobox genes PITX1 and CDX2 are associated with tumor progression and survival in colorectal carcinoma. Int J Colorectal Dis. 2012; 27:1391–9. 10.1007/s00384-012-1460-4 22438068

[20] Miller JR, Moon RT. Analysis of the signaling activities of localization mutants of beta-catenin during axis specification in Xenopus. J Cell Biol. 1997; 139:229–43. 10.1083/jcb.139.1.229 9314542 PMC2139814

[21] Aktary Z, Kulak S, Mackey J, Jahroudi N, Pasdar M. Plakoglobin interacts with the transcription factor p53 and regulates the expression of 14-3-3σ. J Cell Sci. 2013; 126:3031–42. 10.1242/jcs.120642 23687381

[22] Gehmlich K, Lambiase PD, Asimaki A, Ciaccio EJ, Ehler E, Syrris P, Saffitz JE, McKenna WJ. A novel desmocollin-2 mutation reveals insights into the molecular link between desmosomes and gap junctions. Heart Rhythm. 2011; 8:711–8. 10.1016/j.hrthm.2011.01.010 21220045 PMC3085091

[23] Garrod DR, Berika MY, Bardsley WF, Holmes D, Tabernero L. Hyper-adhesion in desmosomes: its regulation in wound healing and possible relationship to cadherin crystal structure. J Cell Sci. 2005; 118:5743–54. 10.1242/jcs.02700 16303847

[24] Sun H, Wang X, Liu K, Guo M, Zhang Y, Ying QL, Ye S. β-catenin coordinates with Jup and the TCF1/GATA6 axis to regulate human embryonic stem cell fate. Dev Biol. 2017; 431:272–81. 10.1016/j.ydbio.2017.09.004 28943339

[25] Hakimelahi S, Parker HR, Gilchrist AJ, Barry M, Li Z, Bleackley RC, Pasdar M. Plakoglobin regulates the expression of the anti-apoptotic protein BCL-2. J Biol Chem. 2000; 275:10905–11. 10.1074/jbc.275.15.10905 10753888

[26] Hoxhaj G, Manning BD. The PI3K-AKT network at the interface of oncogenic signalling and cancer metabolism. Nat Rev Cancer. 2020; 20:74–88. 10.1038/s41568-019-0216-7 31686003 PMC7314312

[27] Janku F, Yap TA, Meric-Bernstam F. Targeting the PI3K pathway in cancer: are we making headway? Nat Rev Clin Oncol. 2018; 15:273–91. 10.1038/nrclinonc.2018.28 29508857

[28] Naderali E, Valipour B, Khaki AA, Soleymani Rad J, Alihemmati A, Rahmati M, Nozad Charoudeh H. Positive Effects of PI3K/Akt Signaling Inhibition on PTEN and P53 in Prevention of Acute Lymphoblastic Leukemia Tumor Cells. Adv Pharm Bull. 2019; 9:470–80. 10.15171/apb.2019.056 31592121 PMC6773944

