# Supplementary material for: DSC2 suppresses the growth of gastric cancer through the inhibition of nuclear translocation of γ-catenin and PTEN/PI3K/AKT signaling pathway
Source: Aging (Albany NY). 2023 Jul 8;15(13):6380–99. doi: 10.18632/aging.204858 (PMC10373986; doi:10.18632/aging.204858)
Supplement: Supplementary Figures [file aging-15-204858-s001.pdf]

SUPPLEMENTARY FIGURES

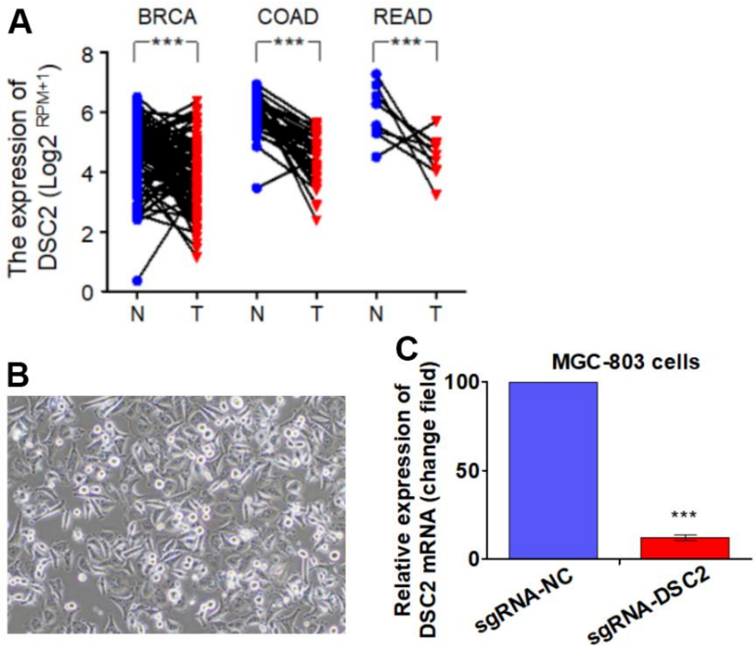

Supplementary Figure 1. DSC2 was knocked down in MGC-803 cells significantly after transfecting with vectors that were inserted into the CRISPR EGFP plasmid. (**A**) Positive clone MGC-803 cells were photoed (100 ×). (**B**) DSC2 gene in sgRNA-NC and sgRNA-DSC2 were detected by qRT-PCR. Data are presented as mean ± SEM from three separate experiments. ***p<0.001 vs. sgRNA-NC. (**C**) Comparison of DSC2 expression of paired samples in breast invasive carcinoma (BRCA), colon adenocarcinoma (COAD), rectum adenocarcinoma (READ), which was collected from the TCGA database. ***p<0.001 vs. Tumor tissues.

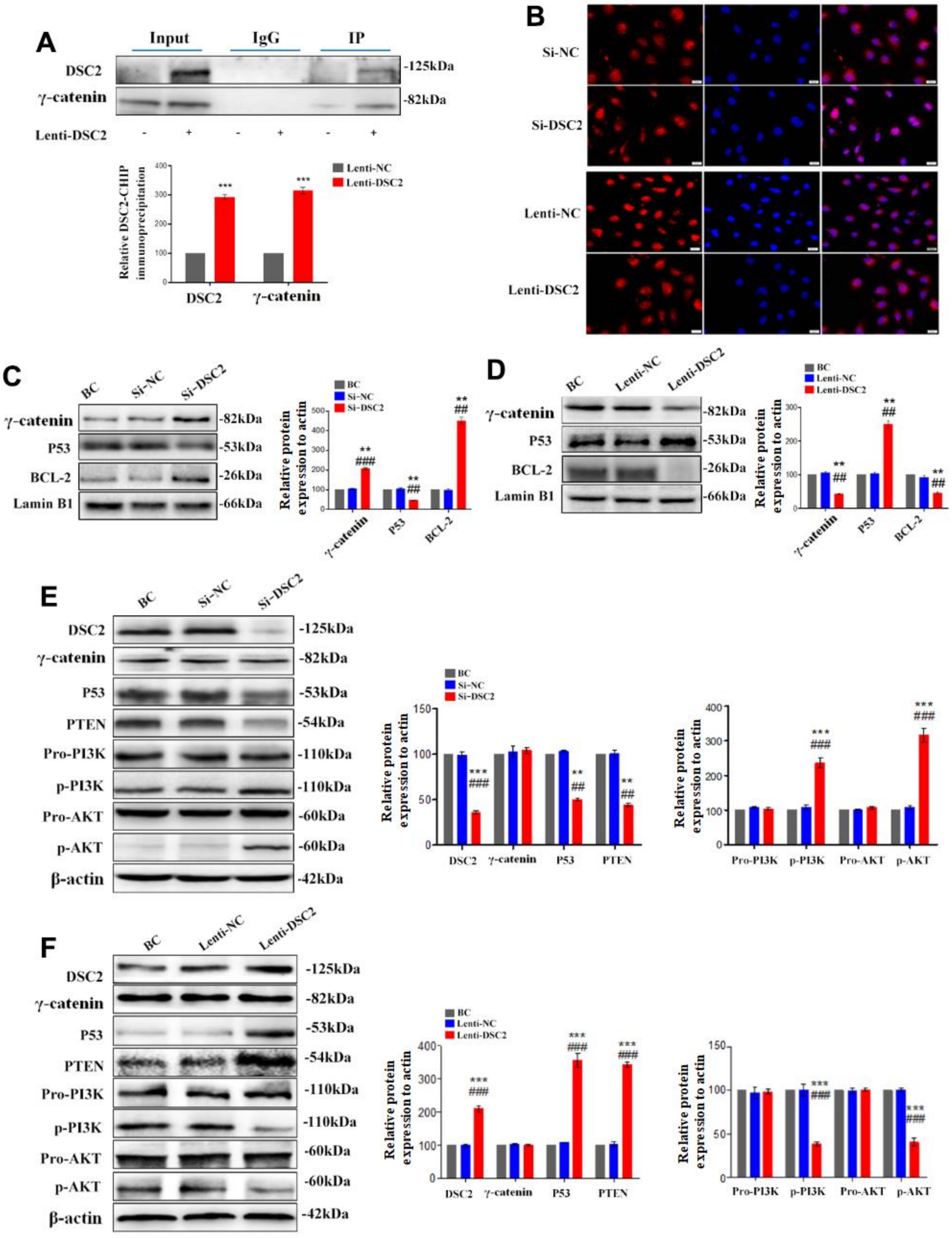

Supplementary Figure 2. DSC2 inhibited the γ-catenin nuclear translocation and suppressed the PI3K/AKT signaling pathway of SGC-7901 cells. (**A**) Co-IP assay was performed to analyze the interaction of DSC2/γ-catenin by DSC2. The data are represented as mean ± SEM, n=3. ***p<0.001 vs. Lenti-NC. After being transfected with siDSC2 or stably expressing DSC2 gene of SGC-7901 cells, (**B**) the level of γ-catenin accumulated in the nucleus was detected by immunofluorescence assay. The scale bar = 20 μm. (**C**) The expression of γ-catenin, BCL-2 and P53 in nucleus was determined by Western blot assay. (**D**) The expressions of γ-catenin, P53, PTEN, pro-PI3K, p-PI3K, pro-AKT, p-AKT were detected by Western blot assay. The data are represented as mean ± SEM, n=3. **p<0.01 and ***p<0.001 vs. BC. ##p<0.01 and ###p<0.001 vs. Si-NC or Lenti-NC group. (**E**, **F**) the expressions of PTEN, pro-PI3K, p-PI3K, pro-AKT, p-AKT were detected by Western blot assay. Data are presented as mean ± SEM from three separate experiments. ***p<0.001 vs. BC. ###p<0.001 vs. Si-NC or Lenti-NC.

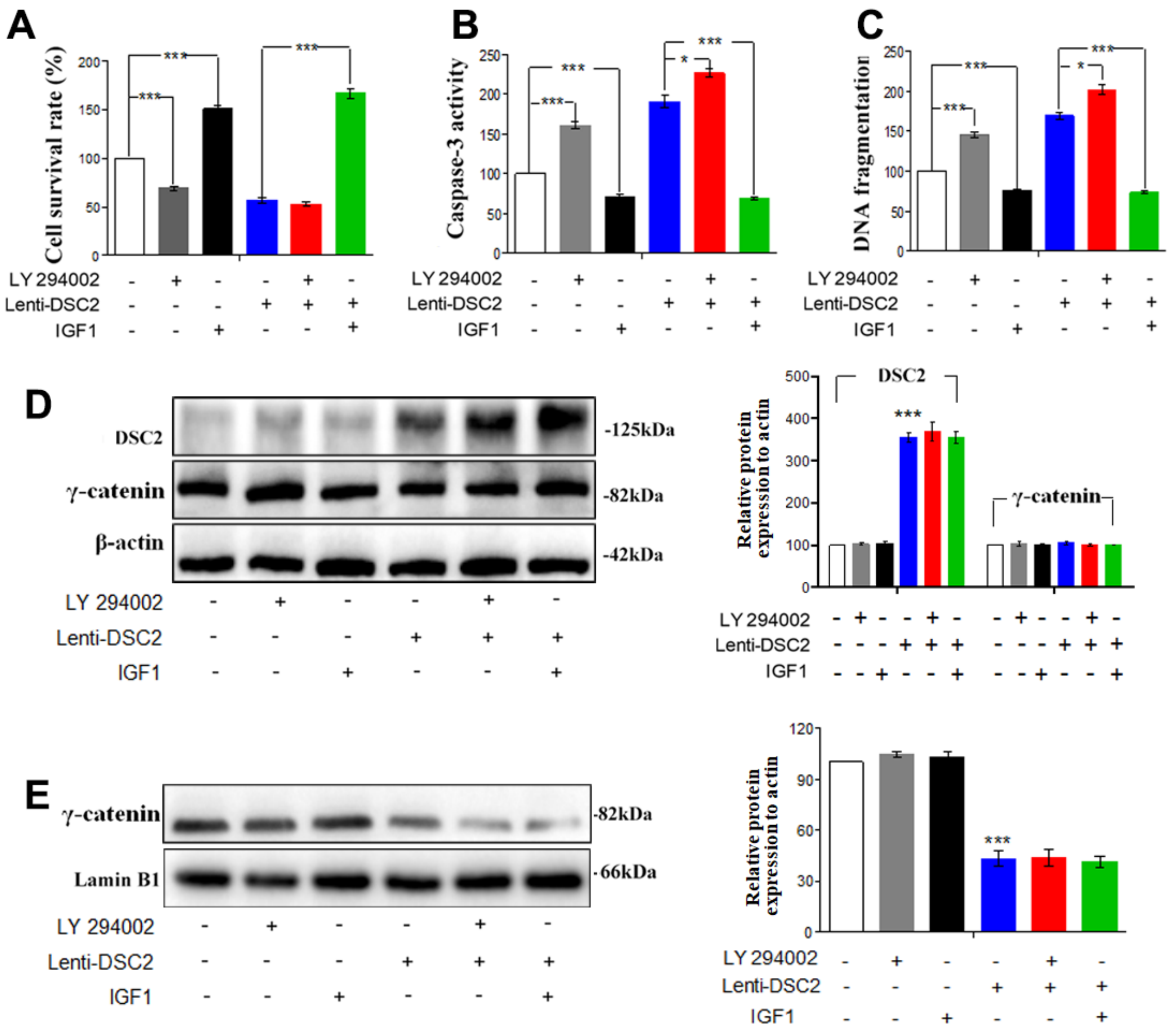

Supplementary Figure 3. DSC2 inhibited viability of SGC-7901 cells through suppressing PI3K/AKT signaling pathway. Effect of DSC2 on the viability of GC cells in the presence of LY294002 and IGF1 was determined by Caspase-3 activity assay (**A**), Sperm DNA fragmentation assay (**B**) and MTT assay (**C**). Data are presented as mean ± SEM from three separate experiments. *p<0.05 and ***p<0.001 vs. Lenti-NC or Lenti-DSC2 group. (**D**, **E**) The levels of γ-catenin both in cells and in the nucleus among SGC-7901 cells that treated with LY294002 or IGF1, were tested by Western blot assay. The data are represented as mean ± SEM, n=3. ***p<0.001 vs. Lenti-NC.
