# Supplementary material for: DSC2 suppresses the growth of gastric cancer through the inhibition of nuclear translocation of γ-catenin and PTEN/PI3K/AKT signaling pathway
Source: Aging (Albany NY). 2023 Jul 8;15(13):6380–99. doi: 10.18632/aging.204858 (PMC10373986; doi:10.18632/aging.204858)
Supplement: Supplementary Table 1 [file aging-15-204858-s002.pdf]

SUPPLEMENTARY TABLE

Supplementary Table 1. Proteomic analysis of proteins linked by DSC2.

Gene name	Protein Name	Peptides (95%)	% Coverage of 95% confidence intervals
DSP	Desmoplakin	48	18
KRT1	Cytokeratin-1	35	60
TFRC	Transferrin receptor protein 1	30	47
KRT9	Keratin, type I cytoskeletal 9	30	65
BRD4	Bromodomain-containing protein 4	29	55
ALB	Albumin	26	43
PLEC	Plectin	20	6
IGF2R	Cation-independent mannose-6-phosphate receptor	20	9
HSPD1	60 kDa chaperonin	20	47
JUP	Junction plakoglobin	19	35
ACAD9	Acyl-Coenzyme A dehydrogenase family, member 9, isoform CRA_b	19	42
PKM	Pyruvate kinase PKM	17	41
HSPA1B	Heat shock 70 kDa protein 1B	16	39
KRAS	KRAS protein	16	25
CTNNA1	Catenin alpha-1	16	22
TRIM21	E3 ubiquitin-protein ligase TRIM21	15	32
LTF	Lactotransferrin	15	26
EEF2	Elongation factor 2	15	22
ANXA2	Annexin A2	15	44
ITGB1	Integrin beta	14	21
HSPA8	Heat shock cognate 71 kDa protein	14	45
LAMP1	Lysosome-associated membrane glycoprotein 1	14	23
HSP90AB1	Heat shock protein HSP 90-beta	14	34
DSG1	Desmoglein-1	14	18
TUFM	Elongation factor Tu	13	28
KRT80	Keratin, type II cytoskeletal 80	13	31
DDX5	DEAD box protein 5	12	29
VIM	Vimentin	11	28
RPS4X	RPS4X protein	11	44
LMNB1	Lamin-B1	11	34
ITGAV	Integrin alpha-V	11	10
DDX3X	ATP-dependent RNA helicase DDX3X	11	22
ATP2A1	Sarcoplasmic/endoplasmic reticulum calcium ATPase 1	11	11
AZGP1	Zinc-alpha-2-glycoprotein	10	30
ALDOA	Fructose-bisphosphate aldolase A	10	34
